# Trends in Alzheimer’s Disease clinical outcome assessments in phase 2 and 3 clinical trials from 1993 to present

**DOI:** 10.1002/alz.089137

**Published:** 2025-01-09

**Authors:** Sarah T Gary, Reina Davis‐Aoki, Prateek Verma, Bryan McDowell

**Affiliations:** ^1^ Clario, Philadelphia, PA USA

## Abstract

**Background:**

Clinical outcome assessments (COAs) are an important part of clinical trials to measure what is meaningful to patients and caregivers. This study aimed to examine trends in Alzheimer’s Disease (AD) COAs used in clinical trials, given the FDA’s recent emphasis on patient‐focused drug development and early AD.

**Method:**

ClinicalTrials.Gov was programmatically accessed through its API on January 19, 2024. Phase 2 and 3 Interventional AD trials were further analyzed for inclusion of 6000+ COAs in the primary or secondary outcome measures field.

**Result:**

997 studies were identified with start dates from 1993‐2025. COAs as primary or secondary outcomes have increased over time (50.0% of studies from 1993‐1999, 57.4% from 2000‐2009, 64.0% from 2010‐2019, 69.1% from 2020‐2023). The increase is mainly driven by an increase in COAs in the primary outcome (40.0% of studies from 1993‐1999 vs. 53.1% from 2020‐2023). ADAS‐Cog was the most common primary COA (19.1% of all studies) followed by CDR (7.0%), CIBIC+(3.3%) and ADCS‐ADL (2.9%). The percentage of studies using ADAS‐Cog as the primary outcome is trending downward, with only 13.0% of studies using ADAS‐Cog as primary in the past 5 years. The MMSE was the most common secondary COA (19.6% of all studies), followed by ADAS‐Cog (16.0%), NPI‐C (15.8%), and ADCS‐ADL (14.5%). Reference to mild cognitive impairment (MCI) in the primary/secondary outcome is trending upward, with no studies in the 90s, 2.2% in the 2000s, 4.6% in the 2010s, and 11.1% from 2020‐2023. In addition, there has been an increase in COAs for preclinical AD and MCI (e.g. PACC, MoCA, iADRS).

**Conclusion:**

Even with the rise in biomarkers and focus on preclinical AD, COAs remain an important part of AD clinical trials, as shown by the continued and increasing usage as primary and secondary outcomes. Historically, ADAS‐Cog has been the gold standard in AD studies, and many approved AD studies have utilized it as primary endpoints with small delta between study drug and placebo. In recent times AD trials are shifting towards MCI‐AD and this has led to new endpoints like PACC and MoCA which are sensitive in identifying early cognitive deficits in patients.

